# Psychosocial Interventions for Pain Management in Advanced Cancer Patients: a Systematic Review and Meta-analysis

**DOI:** 10.1007/s11912-020-0870-7

**Published:** 2020-01-21

**Authors:** Marco Warth, Joshua Zöller, Friederike Köhler, Corina Aguilar-Raab, Jens Kessler, Beate Ditzen

**Affiliations:** 1 Institute of Medical Psychology, Center for Psychosocial Medicine, University Hospital Heidelberg, Bergheimer Str. 20, 69115 Heidelberg, Germany; 2 Psychological Institute, Heidelberg University, Hauptstraße 47-51, 69117 Heidelberg, Germany; 3 Center of Pain Therapy and Palliative Care Medicine, Department of Anesthesiology, University Hospital Heidelberg, Im Neuenheimer Feld 131, 69120 Heidelberg, Germany

**Keywords:** Cancer, Psychotherapy, Music therapy, Relaxation, Pain, Cancer, Palliative care

## Abstract

**Purpose of Review:**

This systematic review and meta-analysis aimed to synthesize the evidence on the effects of psychosocial interventions on pain in advanced cancer patients.

**Recent Findings:**

The included studies investigated the effects of relaxation techniques, cognitive-behavioral therapy, music therapy, mindfulness- and acceptance-based interventions, and supportive-expressive group therapy. Overall, we found a small, but significant effect on pain intensity (*d* = − 0.29, CI = − 0.54 to − 0.05). Effect sizes were highly heterogeneous between studies. We did not find evidence for the superiority of any of the intervention types. However, psychosocial interventions may be more effective if they specifically targeted pain distress as the primary outcome.

**Summary:**

Although findings were mixed, psychosocial interventions can be recommended to complement comprehensive care to alleviate pain in patients facing an advanced or terminal stage of the disease. Future research should develop innovative interventions tailored specifically for pain relief.

## Introduction

Pain is prevalent in 50.7% of cancer patients [1]. In advanced cancer patients and palliative care settings, approximately two in three patients report to be suffering from pain [1–3]. Efficient pain management requires an individualized provision of analgesic medication which has shown to reduce pain levels in the majority of patients [4]. Nonetheless, guidelines and research advocate for a multi-modal treatment of pain in cancer patients combining medical and pharmaceutical expertise with non-pharmacological pain management, e.g., physical therapy and tailored psychosocial approaches [4, 5]. The biopsychosocial model of chronic pain provides a theoretical framework for the necessity of a multi-professional team composition in oncology and palliative care [6–8]. This holistic model takes into account the dynamic interactions between biological, psychological, and social factors in the emergence and maintenance of chronic diseases. For instance, the alienation from normal life due to chronic pain often leads to both a strong dependency on the health care system and withdrawal from social activities. While avoidance behavior may be adaptive in dealing with acute pain, it can be dysfunctional and self-reinforcing in chronic conditions [9].

Not only does the biopsychosocial model predict maladaptive processes, but also it provides potential psychological and social resources for dealing with cancer and the accompanying pain condition. The physical sensation of pain can strongly be shaped by the individual’s psychological appraisal [10]. Hence, the subjective judgment in terms of “How threatening is the pain?” and “How much control do I have over the pain?” has a major effect on how a patient experiences pain which, in turn, influences personal core beliefs and future behaviors [9]. Therefore, psychosocial interventions which specifically establish psychological resources through addressing beliefs and attitudes may support patients in coping with pain.

When it comes to social resources, support from close persons can be a major factor [11]. However, cancer patients may not always be part of a well-functioning social system or their relatives may be burdened themselves by the illness of a loved one. Social strain thus can also lead to reduced psychological well-being in patients having more difficulties to cope with pain. Accordingly, psychosocial interventions may buffer the socially impairing effects of a terminal illness and its treatment.

Based on the perspective of biopsychosocial integration, a wide array of non-pharmacological interventions is available to cancer patients nowadays. Evidence-based approaches encompass physical treatments like massage and gymnastics, educational interventions with respect to illness and treatment, and psychosocial interventions. The latter can play an important role in improving the quality of life of patients, especially in advanced cancer stages or palliative care where physical activity is strongly limited. Psychosocial interventions specifically address feelings, thoughts, and behavior [12], including but not limited to psychotherapy, art and music therapy, cognitive-behavioral interventions, mindfulness-based techniques, relaxation training, supportive-expressive therapy, hypnotherapy, and coping skills strategies.

Generally, psychosocial interventions have a beneficial effect on the quality of life and other psychological health outcomes in cancer patients across all stages of the disease [13, 14]. In recently diagnosed patients, meta-analytic findings showed improvements in cancer-specific quality of life and psychological distress [15]. Other systematic reviews found beneficial effects on quality of life for specific tumor entities, such as gynecological [16, 17] and prostate cancer [18], although authors consistently state that the study quality was low and that findings on secondary outcomes were mixed.

Furthermore, only some of these studies assessed pain as a primary or secondary outcome. Meta-analyses revealed small, but significant effects of psychosocial interventions on pain severity and pain interference in various stages of the disease [19], and for breast cancer patients in particular [20, 21]. (Psycho-)education, hypnosis, cognitive-behavioral approaches, and relaxation/imagery were proposed as potentially effective supportive treatments in the management of pain [12, 22]. Studies often had a high risk of bias and higher study quality was associated with weaker effect sizes [20].

Even fewer research studied patient populations in advanced and terminal cancer stages [23•]. Cognitive-behavioral psychotherapy and mindfulness-based interventions were reported to have beneficial effects on quality of life, depression, and anxiety in advanced cancer patients [24–27]. In a recent meta-analysis by our own group, we found that even very brief psychosocial interventions can improve quality of life and reduce emotional and existential distress in terminally ill patients receiving palliative care [23•].

During the fluent transition to palliative and end-of-life care settings, effective pain management can be a crucial determinant of cancer patients’ quality of life [2, 3]. Considering the multi-professional team composition in palliative care, it is essential to evaluate the various treatment options from a biopsychosocial perspective. Surprisingly, only few clinical trials tested psychosocial interventions for pain treatment in patients with advanced cancer [12], and no meta-analysis has yet summarized the findings of existing studies. Since advanced cancer and palliative care patients’ needs may differ from those of patients in earlier stages, results from reviews with a broader scope on cancer can not necessarily be transferred. For instance, the declining physical condition may restrict the feasibility of certain interventions. Moreover, time constraints due to limited life expectancy demand interventions to be immediately effective. Therefore, it is crucial to know which interventions are most effective in advanced stages. The present review therefore aims to provide an overview of the effects of psychosocial interventions on pain in advanced cancer treatment and palliative care.

## Methods

To summarize the latest evidence on the abovementioned research topic, we conducted a systematic review in accordance with the Preferred Reporting Items for Systematic Reviews and Meta-Analyses (PRISMA) guidelines [28]. Moreover, effect sizes from all eligible primary studies that provided sufficient statistical data were aggregated in the course of meta-analyses.

### Eligibility Criteria

Inclusion criteria were defined according to the PICOS framework (participants, interventions, comparisons, outcomes, study design) [28]. The review focused on adult patients with advanced cancer. For study inclusion, at least 80% of the sample had to be diagnosed with cancer and at least 50% of the sample should consist of stage IV cancer patients. Studies were eligible if they used psychosocial interventions provided by a therapist or nurse as described in the “Introduction” section. The intervention group could be compared with no treatment/waitlist, standard care, or active social support. The outcome measures were pain intensity and pain interference. We included only studies with a control group design and pre- to post-intervention assessments.

### Literature Search

A systematic search was conducted using the electronic databases PubMed, PsycINFO, and CINAHL. We combined search terms describing psychosocial interventions (e.g., psychotherap*) with those targeting at palliative care/advanced cancer, at clinical trials (e.g., random*), and at pain. The complete search syntax can be sent on request by the corresponding author. In addition to the electronic search, the reference lists of the selected studies and similar systematic reviews were hand-searched. The search was conducted in June 2019.

### Study Selection and Data Extraction

We entered records identified through database (*n* = 376) and hand-search (*n* = 3) into Rayyan, an online tool for systematic reviews [29]. After the removal of duplicates, two independent raters screened the abstracts of the remaining records (*n* = 209). Conflicting assessments were resolved by discussion between the two raters. A total of 182 records were excluded because they did not meet the criteria with regard to publication type (*n* = 67), study design (*n* = 68), population (*n* = 22), intervention (*n* = 18) or outcome (*n* = 5), or no full-text access (*n* = 2). Complete full texts of the remaining studies (*n* = 27) were then screened. Of those, 13 were excluded because they did not meet the criteria regarding publication type (*n* = 3), study design (*n* = 1), population (*n* = 3), intervention (*n* = 2), or outcome (*n* = 4). Finally, 14 articles were included in the narrative synthesis. In order to perform a quantitative synthesis, authors were contacted via e-mail asking for data that was not reported, but necessary for the calculations. Three articles were excluded because the data could not be obtained. Eleven articles were included in the quantitative synthesis. We then extracted basic characteristics together with information on patients, interventions, study designs, outcomes, and statistical results according to a predefined data coding sheet.

### Statistical Analysis

Standardized mean change differences for pretest-posttest-control-group designs were calculated following the method described by Morris [30]. In order to calculate the sampling variance, an estimate of the pain pretest-posttest correlation based on the author’s previous research was used [31]. As the calculated effect size is based on Cohen’s *d*, they can be interpreted correspondingly (small: *d* = 0.2–0.5; medium: *d* = 0.5–0.8; large: *d* > 0.8) [32].

For further meta-analytic calculations, we used the metafor package in R [33]. Heterogeneity was tested using *Q*- and *I*^*2*^-statistics. Moderator analyses were conducted to determine the following possible sources of heterogeneity: type of setting (inpatient, outpatient, mixed), type of intervention (creative arts based, cognitive-behavioral interventions, mindfulness and acceptance based, supportive expressive therapy, relaxation and guided imagery), number of sessions, total duration of the intervention, type of control (treatment as usual, active control), and as pain primary vs. secondary outcome. Type-I error probability was set at *α* = 0.05.

## Results

The following section is a narrative synthesis of information from primary studies, categorized for different intervention approaches. Table 1 gives an overview of the study characteristics and main findings.

**Table 1:** Study characteristics

Study	Patients	Intervention	Control group	Pain as target outcome	Main findings on pain outcomes
Arathuzik et al.	Inpatient and outpatient oncological unit, all advanced breast cancer, *N* = 24	1: PMR and imagery; 1 session, 75 min; 2: PMR, imagery, and cognitive coping; 1 session, 120 min	Standard care	Yes	No differences in pain intensity and pain distress between groups; increase in ability to control pain in IG
Butler et al.	Outpatient oncological unit, all breast cancer, *N* = 124	Supportive-expressive group therapy, 52 sessions, 90 min	Education	Yes	Weaker increase in pain intensity in IG
Goodwin et al.	Outpatient cancer center, all breast cancer, *N* = 235	Supportive-expressive group therapy, 52 sessions, 90 min	Social support and education	No	Weaker increase in pain intensity in IG
Gutgsell et al.	Inpatient medical center, advanced cancer, 26 non-cancer patients, *N* = 200	Music therapy; 1 session, 20 min	Waitlist with relaxation	Yes	Stronger decrease in pain intensity in IG
Horne-Thompson and Grocke	Inpatient palliative care, 1 non-cancer patient, *N* = 25	Music therapy; 1 session, 20–40 min	Waitlist with volunteer visit	No	Stronger decrease in pain intensity in IG
Kwekkeboom et al.	Outpatient oncological unit, all advanced cancer, *N* = 86	Cognitive-behavioral intervention; 14 sessions, 20 min	Standard care	No	Stronger decrease in pain intensity in IG
Kwekkeboom et al.	Outpatient oncological unit, all cancer, *N* = 164	Cognitive-behavioral intervention; 21 sessions, 5–25 min	Education	No	No differences in pain intensity between groups
Mosher et al.	Outpatient cancer center, all advanced breast cancer, *N* = 47	Acceptance and commitment therapy; 6 sessions, 60 min	Education + support	No	No differences in pain intensity and pain interference between groups
Porter et al.	Outpatient oncology clinic, all breast cancer, *N* = 63	Mindful yoga; 8 sessions, 120 min	Social support	No	No differences in pain intensity and pain interference between groups
Ramirez et al.	Inpatient palliative care, all advanced cancer, *N* = 40	Music therapy; 1 session, 30 min	Conversation about music	No	No differences in pain intensity between groups
Rodin et al.	Inpatient cancer center, all blood cancer, *N* = 42	Cognitive-behavioral therapy; 8–12 sessions, 30–60 min	Standard care	No	Stronger decrease in pain intensity and pain interference in IG
Warth et al.	Inpatient palliative care, 2 non-cancer patients, *N* = 84	Music therapy; 2 sessions, 30 min	Prerecorded mindfulness exercise	No	No differences in pain intensity between groups
De Paolis et al.	Hospice care, all terminal cancer, *N* = 104	PMR and imagery; 1 session, 20 min	Standard care	Yes	Stronger decrease in pain intensity in IG
Tsai et al.	Inpatient palliative care unit, all advanced cancer, *N* = 37	Breathing and biofeedback; 6 sessions, 45 min	Standard care	Yes	Stronger decrease in pain intensity in IG

*IG* intervention group

### Relaxation and Guided Imagery

Guided relaxation appears to be effective in the treatment of pain [34••, 35, 36] while the findings regarding self-administered relaxation show inconsistencies [37, 38•]. One study examined the efficacy of progressive muscle relaxation (PMR) and interactive guided imagery (IGI) on pain in patients with advanced cancer [34••]. Patients received one session of PMR and IGI where patients were guided to visualize pleasant images. The decrease in pain intensity was significantly stronger for patients in the intervention group than in a standard care control group. In contrast, another study [36] found no differences between intervention and control groups with regard to pain intensity and pain distress. This study examined the efficacy of relaxation and cognitive coping skills training on pain in patients with metastatic breast cancer. Patients in the two intervention groups attended either one session consisting of a PMR exercise followed by IGI or the same exercises complemented by a cognitive coping skills training. The control group received standard care. Despite no differences in pain intensity and pain distress, the ability to control pain was reported to be higher in both intervention groups compared with that in the control group.

While the aforementioned studies explored the effects of live guided relaxation, Kwekkeboom et al. examined the efficacy of self-administered mp3-recorded exercises consisting of relaxation, imagery, and distraction techniques. In a pilot study [37] and a full-scale RCT [38•], advanced cancer patients received an introductory therapeutic session and were encouraged to complete at least 1 of the 12 exercises on their own each day. Two weeks later, pain severity decreased in the intervention group of the pilot study in comparison with the waitlist controls. However, in the full-scale study, no effects on pain could be observed after 3, 6, and 9 weeks in comparison with a control group that listened to recordings of educational material.

Another self-administered relaxation technique is electromyography (EMG) biofeedback–assisted relaxation which Tsai et al. investigated in patients with advanced cancer [35]. In 6 sessions over 4 weeks, patients were instructed to take slow, deep diaphragmatic breaths and to decrease a visual and auditory EMG signal of the frontalis muscle. Patients in the intervention group reported a significantly larger reduction of pain intensity in comparison with the control group receiving usual care.

### Creative Arts-Based Therapies

Although the search terms aimed at a variety of creative arts–based approaches (e.g., arts therapy), only studies regarding music therapy fit the criteria. Other creative arts–based therapies such as dance and movement therapy may not be applicable in palliative settings. The results are inconclusive with some studies providing evidence for music therapy as effective for pain treatment [39, 40] and others not [31, 41, 42]. Horne-Thompson and Grocke found a positive effect of music therapy, as terminally ill patients receiving music therapy reported lower pain than the waitlist control group [40]. In their study, music therapy consisted of one session including various techniques, e.g., singing or relaxation with music. Supporting these findings, Gutgsell et al. also found a greater decrease in pain in the music therapy group compared with the waitlist control group [39]. Music therapy here was composed of one session of autogenic relaxation and the imagination of a safe place accompanied by live music.

In contrast, Warth et al. found no differences in pain perception between the intervention and control groups [31]. Palliative care patients in the music therapy group received two sessions of a live music–based relaxation involving voice and monochord. The control exercise consisted of a prerecorded mindfulness exercise via headphones. Ramirez et al. examined the effect of music therapy on emotional response assessed by EEG and self-reported symptoms including pain in patients with advanced cancer [42]. Patients in the music therapy group participated in one session consisting of songs and a relaxation/imagery exercise, yet no significant differences in pain could be observed compared with the control group that received only company.

### Cognitive-Behavioral Interventions

Cognitive-behavioral interventions can also support pain relief in cancer care. The EASE study by Rodin et al. examined the efficacy of a combination of psychotherapy and physical symptom screening on pain, other symptoms, and quality of life in patients with acute leukemia [43••]. Over 8 weeks, patients received 8–12 psychotherapeutic sessions based on principles of supportive psychotherapy and trauma-focused CBT. When the ratings of one of the symptoms passed a threshold value, patients were referred to early palliative care until the symptoms scores decreased again. Pain intensity and pain interference decreased in the intervention group, while patients in usual care demonstrated an increase in pain interference.

### Mindfulness- and Acceptance-Based Interventions

Acceptance and commitment therapy (ACT) aims at increasing present-moment awareness and psychological flexibility through mindfulness and behavior-change strategies. Therefore, the interference of unwanted internal experiences, like pain, may also be decreased. Mosher et al. examined the efficacy of acceptance and commitment therapy in patients with metastatic breast cancer [44]. Patients received six telephone sessions of ACT dedicated to mindfulness practice and acceptance of thoughts, feelings, and symptoms. In comparison with the control group that received an educational and supportive intervention, there were no observed effects on pain and pain interference. Porter et al. investigated the efficacy of mindful yoga on pain and other symptoms in patients with metastatic breast cancer [45]. The 8 weekly group sessions of yoga consisted of physical postures, breathing exercises, meditation techniques, and group discussions. Again, there were no significant differences in pain-related measures compared with the control group that received social support in the same time frame.

### Supportive-Expressive Group Therapy

Two studies with the same design showed that supportive-expressive group therapy in combination with hypnosis reduces the increase of pain experience in patients with metastatic breast cancer over a time period of 1 year [46, 47]. Patients received weekly sessions with varying group size and varying total amount of sessions. The aim of the intervention was to create a supportive environment for the patients to be able to confront their problems and encourage the expression of emotions. Each session ended with a hypnosis exercise intended to alter the sensation in a part of the body that hurt. In both studies, after 12 months of ongoing intervention, patients reported a significantly smaller increase in pain experience than the control group that received education material only.

### Pooled Effects of Psychosocial Interventions on Pain Intensity and Pain Interference

**Fig. 1:**
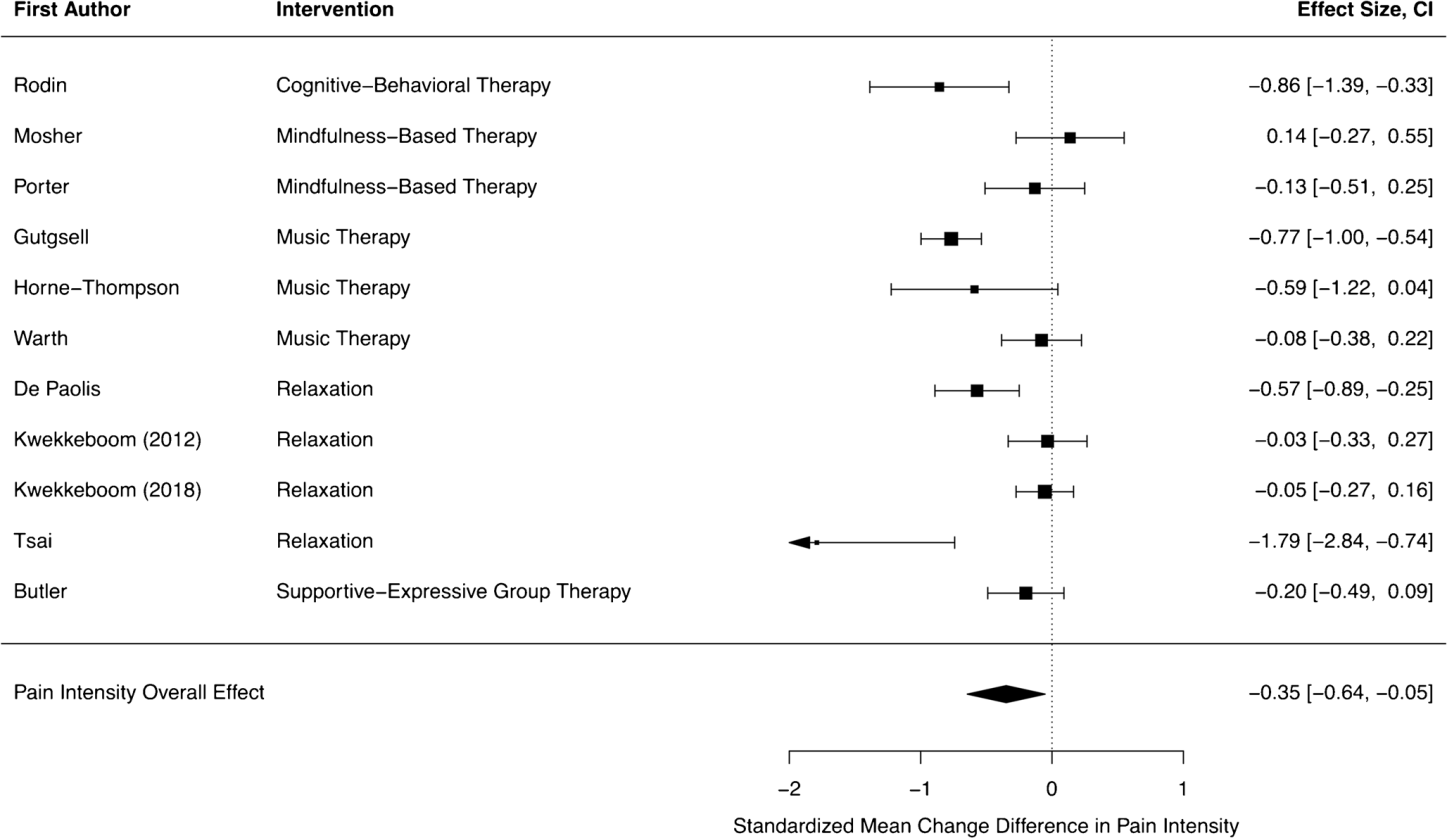
Forest plot for pain intensity. CI, 95% confidence interval

Eleven studies were included in the quantitative synthesis. All of them provided data on pain intensity and three additionally reported data on pain interference. A random effects model revealed a small, but statistically significant reduction of pain intensity with a pooled effect of *d* = − 0.35 (CI = − 0.64 to − 0.05, *p* = 0.03) and significant heterogeneity among the individual effects (*Q* = 46.90, *p* < 0.01, *I*^2^ = 79%). Figure 1 shows all effect sizes from the primary studies together with the pooled effect.

Visual inspection of the individual effects revealed one possible outlier [35] with a particularly strong effect, but low weight. The overall effect remained robust after exclusion of this study in the course of sensitivity analysis (*d* = − 0.29, CI = − 0.54 to − 0.05). None of the tested moderators could significantly help to explain variance between primary effect sizes (all *p* > 0.05). However, the distinction between studies that specifically targeted pain as the primary outcome and those who did not, revealed a statistic trend (*p* = 0.09). This finding suggests that pain-specific psychosocial treatments may lead to greater pain reductions (*d* = − 0.44) than interventions that primarily aimed at different constructs, such as depression, quality of life, or general symptom distress (*d* = − 0.17).

Regarding pain interference, the pooled effect was weak and not statistically significant (*d* = − 0.28, CI = − 1.69 to 1.12, *p* = 0.48). The overall effect is presumably heterogeneous, but because of the small number of individual effects, no further analyses were performed.

## Discussion

### Summary and Interpretation of Results

The present study aimed to provide an overview of the effects of psychosocial interventions on pain in advanced cancer treatment and palliative care. For this purpose, we conducted a systematic review and meta-analysis. Overall, we found a beneficial, but heterogeneous effect with some inconsistent results between and among the different intervention categories.

Relaxation techniques, in general, showed a promising potential in improving symptom distress by pain in advanced cancer patients, although studies showed some inconsistencies. Our descriptive analyses suggest that personally guided techniques [34••, 35, 36] may be more effective than self-administered relaxation [37, 38•]. One possible explanation is that the effort of self-administration itself may bind attention that could make it more difficult to focus on the actual relaxation. However, the latter set of studies also aimed at broader outcomes (e.g., general symptom distress) and was not specifically tailored to induce pain relief. Moreover, methodological factors such as longer intervention time spans may also produce heterogeneity in the results.

Among creative arts–based therapies, only studies with music therapy met the inclusion criteria. Two studies supported its efficacy with regard to pain [39, 40], while other studies did not observe any effects [31, 42]. For Warth et al. [31], the lack of significant findings may be related to low baseline scores. In contrast to Gutgsell et al. [39], the authors did not define pain as the primary outcome and hence, did not screen for patients with initially high pain ratings. Moreover, the effects for music therapy on pain were stronger when compared with waitlist control groups [39, 40] in contrast to active control groups, like prerecorded mindfulness or company [31, 42]. Future studies might consider three-arm study designs to gain further insights on the importance of the control group in this context.

One recent trial was categorized as primarily cognitive-behavioral [43••]. Both pain intensity and pain interference decreased in the psychotherapeutic intervention group over the 8-week study course, suggesting CBT might be a promising long-term psychosocial intervention for pain treatment.

When it comes to mindfulness- and acceptance-based interventions, the two included studies did not provide evidence for its efficacy on pain in advanced cancer patients [44, 45]. One reason could be the lack of in-person implementation of the intervention: In Mosher et al.’s study [44], the Acceptance and Commitment Therapy was conducted via telephone. Similarly, participants in Porter et al.’s study [45] received a group intervention which might have weaker effects than individual sessions with more personal attention, especially when compared with a social support control group. Nevertheless, since mindfulness-based interventions have proven beneficial in other studies with cancer patients and for other outcomes [48], further research with advanced cancer patients can be highly encouraged.

Finally, both studies investigating supportive-expressive group therapy in combination with hypnosis showed beneficial effects for pain intensity in breast cancer patients [46, 47]. Notably, as the intervention lasted for 12 months, it may mainly be recommended for patients with a longer life expectancy or as an early palliative care treatment option.

In general, the data shows that interventions from only five studies were explicitly targeting pain as the primary outcome. It is reasonable to assume that this will substantially determine the efficiency, which was at least partly supported by our moderator analysis.

### Limitations

The methodology of the present review itself faces several limiting factors. First, the number of identified primary studies was relatively low. Despite the fact that we tried to contact authors repeatedly and via multiple communication channels, we were not able to receive sufficient data from three identified studies, which additionally limits the generalizability of the meta-analytic findings. Second, the included studies were highly heterogeneous with respect to session frequency, intervention type and setting, sample composition, pain measures, and study quality. Still, possibly due to the low number of studies, we could not further explain heterogeneity in the moderator analyses. Finally, we decided to include only short-term effects (pre- to post-intervention) in the statistical analyses, as only four studies implemented and reported follow-up data assessments. Hence, no conclusions about long-term effects of psychosocial interventions can be drawn from this overview.

## Conclusions

The present review shows that psychosocial interventions, in general, can have a beneficial effect on self-rated pain severity of advanced cancer patients. Thus, psychosocial interventions can be recommended to complement state-of-the-art medical pain control. Adverse effects are rarely reported, and psychosocial cancer care may additionally have a preventive effect on other common symptoms, including fatigue, depression, and anxiety. While the magnitude of effects did not vary for different intervention types, we identified relaxation-based techniques, music therapy, cognitive-behavioral therapy, and supportive-expressive group therapies as promising. However, findings for all intervention types were mixed and more research is needed on techniques that are specifically designed to target pain symptoms.
