# Correction to: Psychosocial Interventions for Pain Management in Advanced Cancer Patients: a Systematic Review and Meta-analysis

**DOI:** 10.1007/s11912-021-01063-5

**Published:** 2021-04-22

**Authors:** Marco Warth, Joshua Zöller, Friederike Köhler, Corina Aguilar-Raab, Jens Kessler, Beate Ditzen

**Affiliations:** 1 Institute of Medical Psychology, Center for Psychosocial Medicine, University Hospital Heidelberg, Bergheimer Str. 20, 69115 Heidelberg, Germany; 2 Psychological Institute, Heidelberg University, Hauptstraße 47-51, 69117 Heidelberg, Germany; 3 Center of Pain Therapy and Palliative Care Medicine, Department of Anesthesiology, University Hospital Heidelberg, Im Neuenheimer Feld 131, 69120 Heidelberg, Germany

**Correction to: Current Oncology Reports (2020) 22: 3**

10.1007/s11912-020-0870-7

The article “Psychosocial Interventions for Pain Management in Advanced Cancer Patients: a Systematic Review and Meta-analysis”, written by Marco Warth, Joshua Zöller, Friederike Köhler, Corina Aguilar-Raab, Jens Kessler, and Beate Ditzen, was originally published Online First without Open Access. After publication in volume 22, issue 1, article 3 the author decided to opt for Open Choice and to make the article an Open Access publication. Therefore, the copyright of the article has been changed to © The Author(s) 2021 and the article is forthwith distributed under the terms of the Creative Commons Attribution 4.0 International License, which permits use, sharing, adaptation, distribution and reproduction in any medium or format, as long as you give appropriate credit to the original author(s) and the source, provide a link to the Creative Commons licence, and indicate if changes were made. The images or other third party material in this article are included in the article’s Creative Commons licence, unless indicated otherwise in a credit line to the material. If material is not included in the article’s Creative Commons licence and your intended use is not permitted by statutory regulation or exceeds the permitted use, you will need to obtain permission directly from the copyright holder. To view a copy of this licence, visit http://creativecommons.org/licenses/by/4.0/.

